# The Role of Behavioral Science in Personalized Multimodal Prehabilitation in Cancer

**DOI:** 10.3389/fpsyg.2021.634223

**Published:** 2021-02-16

**Authors:** Chloe Grimmett, Katherine Bradbury, Suzanne O. Dalton, Imogen Fecher-Jones, Meeke Hoedjes, Judit Varkonyi-Sepp, Camille E. Short

**Affiliations:** ^1^School of Health Sciences, University of Southampton, Southampton, United Kingdom; ^2^School of Psychology, University of Southampton, Southampton, United Kingdom; ^3^Survivorship and Inequality in Cancer, Danish Cancer Society Research Center, Copenhagen, Denmark; ^4^Department of Clinical Oncology and Palliative Services, Zealand University Hospital, Næstved, Denmark; ^5^Perioperative Medicine, University Hospital Southampton NHS Foundation Trust, Southampton, United Kingdom; ^6^Department of Medical and Clinical Psychology, CoRPS—Center of Research on Psychological and Somatic Disorders, Tilburg University, Tilburg, Netherlands; ^7^NIHR Southampton Biomedical Research Center, University Hospital Southampton, Southampton, United Kingdom; ^8^Faculty of Medicine, Dentistry, and Health Sciences, Melbourne School of Psychological Sciences, The University of Melbourne, Melbourne, VIC, Australia; ^9^Faculty of Medicine, Dentistry, and Health Sciences, Melbourne School of Health Sciences, The University of Melbourne, Melbourne, VIC, Australia

**Keywords:** prehabilitaion, behavior change, behavioral science, co-design, interventions, oncology, cancer

## Abstract

Multimodal prehabilitation is increasingly recognized as an important component of the pre-operative pathway in oncology. It aims to optimize physical and psychological health through delivery of a series of tailored interventions including exercise, nutrition, and psychological support. At the core of this prescription is a need for considerable health behavior change, to ensure that patients are engaged with and adhere to these interventions and experience the associated benefits. To date the prehabilitation literature has focused on testing the efficacy of devised exercise and nutritional interventions with a primary focus on physiological and mechanistic outcomes with little consideration for the role of behavioral science, supporting individual behavior change or optimizing patient engagement. Changing health behavior is complex and to maximize success, prehabilitation programs should draw on latest insights from the field of behavioral science. Behavioral science offers extensive knowledge on theories and models of health behavior change to further advance intervention effectiveness. Similarly, interventions developed with a person-centered approach, taking into consideration individual needs and preferences will increase engagement. In this article, we will provide an overview of the extent to which the existing prehabilitation literature incorporates behavioral science, as well as studies that have explored patient's attitudes toward prehabilitation. We will go on to describe and critique ongoing trials in a variety of contexts within oncology prehabilitation and discuss how current scientific knowledge may be enhanced from a behavioral science perspective. We will also consider the role of “surgery schools” and detail practical recommendations that can be embedded in existing or emerging clinical settings.

## Introduction

Despite advancements in cancer therapies and surgical techniques 15–40% of cancer patients who undergo surgical treatment experience postoperative complications (Hughes et al., 2019). This can lead to increased hospital stay, hospital readmissions and detrimental effects on quality of life, physical functioning and psychosocial outcomes (Durrand et al., 2019). Multimodal prehabilitation is increasingly recognized as an important component of the pre-operative pathway in oncology. It aims to optimize physical and psychological health through delivery of a series of tailored interventions including exercise, nutrition, and psychological support.

Historically evaluations of the efficacy of prehabilitation have focused on physiological outcomes and physiological mechanisms of action. However, multimodal prehabilitation programs require significant patient engagement. Firstly, patients must choose whether to participate and then engage with multifactorial behavior change in order to adhere, for example, to exercise regimes and dietary changes.

Changing health behaviors is complex and requires much more than provision of information. Interventions that seek to support individual behavior change are most effective when they draw on behavioral science (National Institute of Clinical Excellence (NICE), 2014). Furthermore, interventions developed with a person-centered approach, considering individual needs and preferences will increase patient engagement and are more effective than expert only design processes (Trischler et al., 2018).

This paper describes the existing evidence on patient experience and attitudes toward prehabilitation. We illustrate how inclusion of behavioral science could strengthen uptake and adherence to prehabilitation programs, as well as current evidence of integration of this discipline in the field, both in research and clinical settings.

## Modes of Prehabilitation Delivery and Patient Experience

The optimal mode for providing interventions to enhance physical and psychosocial wellbeing of people with cancer continues to be debated.

Supervised in-person programs delivered via health professionals are arguably considered the gold standard in terms of safety and efficacy (Cormie et al., 2018; Newton et al., 2018). Furthermore, there is evidence that those who are willing and able to participate in such programs experience significant benefits beyond physiological optimization, such as improvements in quality of life, cultivating a positive attitude and fostering a strong sense of purpose (Burke et al., 2013). However, delivery costs are prohibitive, and few programs are available (Dennett et al., 2017). There is also consistent evidence that cancer patients face barriers to attending in-person supervised programs. These include transportation, parking and time, as well as a desire to avoid additional hospital appointments (Ferreira et al., 2018). Some cancer patients express a preference for flexible home-based programs (Hardcastle and Cohen, 2017) however a study exploring the experiences of such a program recounts some patients felt a greater involvement from health care professionals would increase engagement, particularly if they were lacking “energy” or “willpower” (Beck et al., 2020). The fundamental issue spurring the debate is that no one delivery mode offers a program that is effective, safe, person-centered, and widely accessible.

Given the inherent challenges with all approaches, we argue that debating the optimal delivery mode is a moot, counterproductive activity. Rather, attention should be paid to how the limitations of any delivery mode can be addressed, so that programs that best suit the local context can be provided. Many prehabilitation trials described below addressed this by offering a hybrid program, combining supervised sessions and home-based elements. Community based programs that offer more locally available support have also shown positive preliminary results (Loughney et al., 2019). Furthermore, the use of technology is increasing and will likely help to address benefit gaps with distance/home-based programs. In the field of cardiac rehabilitation for example, an approach utilizing sensors and a mobile application to provide real-time supervision of aerobic activity in the local environment was non-inferior to a standard in-clinic approach, was cheaper to deliver and resulted in longer-term behavior change (Maddison et al., 2019).

As a result of the Covid-19 pandemic we will likely see rapid advances in remote delivery of cancer-specific interventions. Prehabilitation clinical teams have responded with agility and adapted programs to online delivery modes. For example, the St Georges Get Set 4 Surgery program (St George's University Hospital NHS Foundation Trust, 2020) used a battery of short videos to continue providing information and advice to their patients. The Perioperative Team at University Hospital Southampton NHS Foundation trust were forced to pause a large prehabilitation randomized controlled trial (Wessex Fit-4-Cancer Surgery trial) (ClinicalTrials.gov, 2018) and developed the SafeFit Trial, which consists of a multi-modal intervention delivered virtually by video conferencing and telephone support (ClinicalTrials.gov, 2020). These changes in service delivery present unique opportunities to add to the evidence-base regarding remote delivery of cancer-specific prehabilitation.

Ultimately, delivery mode decisions will depend on local context and should be based on a needs analysis and consultation with all relevant stakeholders, including the end users. This is exemplified by Tang et al., in their co-design of a prehabilitation service for prostate cancer patients (Tang et al., 2020) and the Manchester Prehab4Cancer clinical service (Moore et al., 2020).

## The Role of Theories and Frameworks of Behavior Change

Once the mode of program delivery has been determined attention can move to identifying the “active ingredients” or individual program components required to meet its objectives. Program developers will have a number of key questions such as:

- How can we encourage uptake to prehabilitation? Especially among patients who stand to benefit the most.
- What are the implications for trying to change multiple health behaviors at once? Should behaviors be changed sequentially (if time allows) or simultaneously?
- How can we promote longer-term behavior changes that will assist with recovery after the operation and reduce the risk of further health issues?

Behavioral scientists are trained in behavioral analysis and the application of intervention planning frameworks like intervention mapping (Bartholomew et al., 1998) which can facilitate this process of intervention development. Like interventions developed within medicine, at the core of such frameworks are: (1) the identification of determinants of the outcome and (2) the identification and application of strategies that effectively target these determinants. Theories of behavior change can help identify appropriate determinants of behavior and strategies to influence those behaviors.

These strategies or “active ingredients” are often referred to as behavior change techniques (BCTs) defined as “an observable, replicable, and irreducible component of an intervention designed to alter or redirect causal processes that regulate behavior” p23 (Michie et al., 2013). Examples include goal setting, graded tasks (set easy to perform goals that get increasingly difficult until the behavior is achieved) and self-monitoring (a method to monitor and record behavior). Michie et al. (2013) developed a Taxonomy of Behavior Change Techniques providing a common language to describe approaches to support behavior change and facilitate synthesis of evidence to support the design of future interventions. Theories and frameworks of behavior change and empirical evidence can guide identification of the most effective BCTs to address the relevant processes that regulate behavior (determinants), which will vary depending on the ambitions of the program and the characteristics of participants.

A discussion of relevant theories and research evidence for addressing prehabilitation objectives is beyond the scope of this article. Rather, it is our intention to highlight that these questions are the remit of behavioral scientists, and to showcase what integration of this expertise into practice might look like. As with all multidisciplinary teams, there can be tensions between disciplines. For example, the clinical team may be focused on the optimal intervention or stimulus for increasing cardiorespiratory fitness prior to surgery, whereas the behavioral expert may prioritize the optimal intervention to maximize motivation. Furthermore, if an ambition of the program is to promote longer-term behavior change patients need to develop skills to engage in these behaviors autonomously. This may be at odds to a highly supervised and structured approach that may be favored by others in the team. By working together, an appropriate balance can be achieved and ultimately enhance program effectiveness.

Relevant input from a behavioral scientist in this context may include integration of strategies for enhancing autonomy, competence, and control within the prescribed program. This could include for example, allowing choices where possible, setting graded tasks, and in the context of exercise, prescribing affect-regulated exercise [i.e., an intensity that feels good (Parfitt et al., 2012)]. These strategies should enhance enjoyment of the program and in doing so increase the likelihood of on-going behavior change (Teixeira et al., 2012). Incorporating strategies to promote habit formation could also help to achieve longer-term outcomes (Gardner et al., 2012). Furthermore, a psychological determinant of behavior change that has received considerable attention is self-efficacy. Defined as “ the belief in one's capabilities to organize and execute the courses of action required to produce given attainments,” self-efficacy has been established as one of the most consistent predictors of adoption and maintenance of physical activity behavior (van Stralen et al., 2009). Studies exploring patient perceptions and experience of prehabilitation programs describe high motivation to engage but confidence to do so as low, hindering engagement (McDonald et al., 2019; Beck et al., 2020). As such, methods to increase self-efficacy to engage in the behaviors required of prehabilitation programs is likely to improve uptake and action. With training, exercise professionals delivering the interventions can incorporate these approaches throughout the program thus maintaining fidelity of the exercise “dose” whilst increasing patient empowerment.

In addition to empirical evidence and theories of behavior change to guide intervention development it is crucial that patients are consulted, thus maximizing engagement and implementation. Several frameworks are available to support such co-creation, including the Person-Based Approach (Yardley et al., 2015). Developed by international leaders in the field of behavioral science, central to the Person-Based Approach is ensuring the needs of the end users are understood and incorporated. This is achieved by using iterative qualitative research (such as interviews and focus groups) at every stage of intervention development and implementation, allowing identification of the key barriers and facilitators to engagement and BCTs to address these (Yardley et al., 2015). Adopting such an approach will help ensure the final program is salient, persuasive, relevant, and achievable for patients.

**Figure 1:**
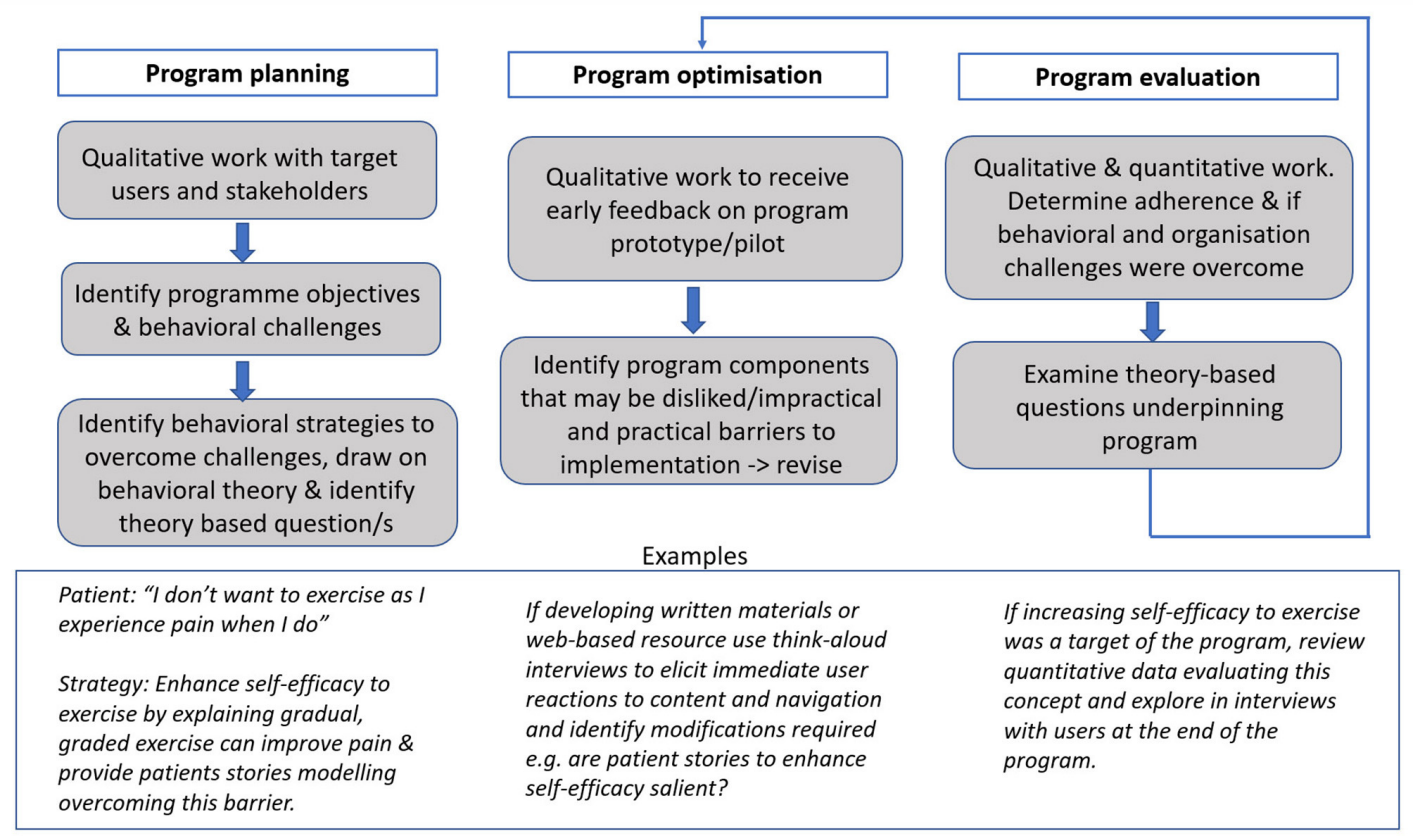
Embedding behavioral science in program planning, optimization, and evaluation.

Working alongside clinical colleagues, a behavioral scientist is well-placed to employ intervention mapping processes, behavioral analysis and patient-centered intervention development. They can also provide training to colleagues delivering the programs to ensure the identified BCTs embedded within it are employed appropriately. This is vital to the integrity of the intervention. For example, when using the BCT of goal setting the process must be collaborative and supportive, enabling the patient to identify salient goals that are meaningful to them. If the goals are directed by the health care professional without appropriate active listening to the patient's needs and circumstance the process is unlikely to be effective. See Figure 1 which illustrates the key principles of behavioral science that can be embedded in the planning, optimisation and evaluation of prehabilitation programs.

## Recent and Ongoing Prehabilitation Trials

In 2018, a comprehensive review of prehabilitation trials was undertaken to inform the development of the Principles and Guidance for Prehabilitation (Macmillan Cancer Support., 2019). As described by Copeland et al. (2020) interrogation of this literature revealed a paucity of consideration of behavioral science. No studies explicitly describe components of the intervention as per the BCT taxonomy. However, in a minority of studies strategies to enhance intervention compliance were included, for example using self-monitoring strategies. However, any behavior change support was poorly described, and none specified the underlying behavioral determinants being targeted.

Examining the literature published since 2018 paints an evolving picture (see Table 1). While many recent and ongoing trials do not include reference to behavior change support there are notable exceptions. Barberan-Garcia et al. (2020) describe a personalized program to promote physical activity in moderate-to-high risk lung cancer patients undergoing thoracic surgery. They report inclusion of behavior change strategies including self-monitoring; comparison of behavior with goal; a daily motivational message; positive reinforcement once a goal is achieved; and provision of educational material. In addition, the cognitive behavioral therapy included in the program aims to “reinforce patients' motivation. and to foster patients' engagement for healthy lifestyles” p. 4 (Barberan-Garcia et al., 2020). Furthermore, in an ongoing trial described by McCourt et al. (2020) the role of behavioral science is explicitly described. This study will investigate the feasibility of exercise-based prehabilitation prior to stem-cell transplantations in myeloma patients. Strategies to promote adherence to the intervention and to change exercise behavior are described as per the BCT taxonomy. Similarly, Macleod et al. (2018) report results of a feasibility trial among adults with stage I–III colorectal cancer. The TreatWELL intervention targeted smoking, alcohol, physical activity, diet, and weight management. The authors describe behavioral approaches informed by self-regulatory theory and the health action process approach. Additionally, the behavior change wheel (a synthesis of 19 behavior change frameworks) (Michie et al., 2011) was used to identify BCTs to motivate and support lifestyle change.

**Table 1:** Recent^a^ and ongoing prehabilitation studies in cancer care: role of behavioral science.

**Authors, (Year) protocol or research, Country**	**Study sample**	**Groups**	**Aim**	**Intervention**	**Role of behavioral science^b^**
Barberan-Garcia et al. (2020) *Study protocol* Spain	Moderate-to-high risk lung cancer patients candidates for thoracic surgery Target sample 158 patients in each group	*Intervention:* Standard preoperative management + personalized multimodal prehabilitation program *Control group:* standard care	To evaluate the cost-effectiveness of a multimodal prehabilitation program supported by information and communication technologies in moderate-to-high risk lung cancer patients undergoing thoracic surgery.	Supervised exercise training program + personalized program to promote physical activity (pedometer and mobile app) Nutritional optimization program (personalized dietary counseling + mobile app) Smoking cessation program (cognitive behavioral intervention + pharmacological therapy) Cognitive behavioral therapy (weekly group sessions)	Not explicitly described. Personalized program to promote physical activity included the following: self-monitoring; comparison of behavior with goal; daily motivational message; positive reinforcement once goal is achieved; provision of educational material. BCTs: self-monitoring of behavior, prompts/cues, discrepancy between behavior and goal, prompts/cues, social reward. Aims of cognitive behavioral therapy: reinforce patients “*motivation; to provide coping strategies to manage stress; to foster patients*” engagement for healthy lifestyles. BCT: social support (unspecified)
Brahmbhatt et al. (2020) *Research article* Canada	22 women undergoing breast cancer surgery	Single group received home-based exercise prehabilitation	Examine feasibility and acceptability of home-based prehabilitation prior to breast cancer surgery and exploration of benefits to physical fitness and patient reported outcomes	Individualized exercise prescription including resistance and mobility training 2–3 days per week and aerobic exercise 30–40 min 3–5 times per week	Not explicitly described Participants received an exercise manual and weekly phone calls or emails to support program compliance. BCT: prompts/cues
Loughney et al. (2019) *Research article* Ireland/UK	24 patients: 14 prostate; 10 colorectal cancer	N/A	To assess compliance and adherence of a pragmatic community-based preoperative exercise program and its effect on health-related components of fitness and HRQoL.	MedEx (ExWell Medical), an established medically supervised chronic illness rehabilitation program delivered in a leisure center.	Not explicitly described.
Macleod et al. (2018) *Research article* UK	22 adults with stage I–III colorectal cancer	N/A	To assess the feasibility of delivering and evaluating a lifestyle program for patients with colorectal cancer undergoing potentially curative treatments.	The TreatWELL intervention program targeted smoking, alcohol, physical activity, diet, and weight management. It was delivered in three face-to-face counseling sessions (plus nine phone calls) by lifestyle coaches over three phases (1: presurgery, 2: surgical recovery, and 3: post-treatment recovery).	The behavioral approaches were informed by two main theoretical frameworks: self-regulatory theory and the health action process approach. Informed by behavior change techniques used in previous interventions and the behavior change wheel, a range of evidence-based behavioral techniques were employed to motivate and support lifestyle change. These included motivational interviewing, implementation intentions, self-monitoring, personalized action and coping plans, feedback, and reinforcement. BCTs: social support (unspecified), action planning, self-monitoring of behavior, problem solving, feedback on behavior, and social reward.
Ngo-Huang et al. (2019) *Research Article* USA	50 patients with resectable pancreatic adenocarcinoma	N/A, single group	To investigate relationships among physical activity, changes in physical function, and health-related quality of life among patients with pancreatic adenocarcinoma enrolled in a home-based exercise prehabilitation program.	Home-based, multimodal exercise program throughout preoperative therapy. All participants met with a registered dietitian, who provided individualized nutrition recommendations.	Not explicitly described. Participants were called by study staff a minimum of once every 2 weeks to encourage adherence. Participants completed daily exercise logs. BCTs: social support (unspecified), self-monitoring of behavior
van Rooijen et al. (2019a) *Study protocol* The Netherlands, Canada, Denmark, France, Italy, Spain	714 patients undergoing colorectal surgery for cancer	Intervention: 4 weeks of prehabilitation Control group: usual care, no prehabilitation	To determine the impact of multimodal prehabilitation on patients' functional capacity and postoperative complications.	Prehabilitation program composed of four elements: exercise training, nutritional intervention, smoking cessation, and psychological support.	Not explicitly described. Participants were phoned weekly to encourage adherence. BCT: social support (unspecified)
Janssen et al. (2020) *Research article* The Netherlands	627 aged ≥70 years who underwent elective surgery for abdominal aortic aneurysm or colorectal cancer	Intervention (*n* = 267): Prehabilitation program Control (*n* = 360): Usual care	To assess the effects of prehabilitation on 1-year mortality and of postoperative delirium and functional outcomes.	Exercise: Unsupervised, home-based personalized resistance and endurance exercises Nutrition: Dietary advice; vitamin supplements, and protein drinks were provided if needed Prevention of delirium: Supplementary interventions to prevent delirium during admission were provided and advice was given on additional preventive measures.	Not explicitly described.
Barrett-Bernstein et al. (2019) *Research article* Canada	172 patients with nonmetastatic colorectal cancer awaiting curative resection	Prehabilitation group vs. control group: rehabilitation	The primary objectives were to (a) assess differences in functional performance and functional capacity and (b) explore the impact of prehabilitation on functional capacity in individuals with depressive symptoms vs. those without.	Moderate-intensity exercise, nutrition therapy, and stress-reducing strategies.	Not explicitly described
Liu et al. (2020) *Research article* China	73 patients undergoing video-assisted thoracoscopic surgery lobectomy for non-small cell lung cancer.	Prehabilitation group (*n* = 37) vs. Usual clinical care control group (*n* = 36)	To investigate the impact of a short-term, home-based, multimodal prehabilitation program on perioperative functional capacity.	2-week home-based, multimodal intervention program before surgery, including aerobic and resistance exercises, respiratory training, nutrition counseling with whey protein supplementation, and psychological guidance.	Not explicitly described. Patients completed diaries to note activities performed. Patients received an instruction booklet and a physical therapist demonstrated resistance training exercises. BCT: self-monitoring of behavior, demonstration of behavior.
Minnella et al. (2019) *Research article* Canada	70 adult patients scheduled for elective radical cystectomy for nonmetastatic bladder cancer	Prehab group (*n* = 35): multimodal prehabilitation Control group (*n* = 35): standard care	To determine whether a preoperative multimodal intervention is feasible and effective in radical cystectomy.	Preoperative multimodal intervention including aerobic and resistance exercise (individualized, home-based moderate-intensity aerobic and resistance activity), diet therapy, and anxiety-reducing intervention (relaxation techniques).	Not explicitly described. Participants were provided with a logbook to record activities. BCT: self-monitoring of behavior
Bousquet-Dion et al. (2018) *Research article* Canada	Patients scheduled for non-metastatic colorectal cancer resection	PREHAB+ (*n* = 41) standard prehabilitation + weekly supervised exercise session vs. REHAB (*n* = 39) standard rehabilitation program	To determine whether a weekly supervised exercise session could provide further benefit to the current prehabilitation program, when comparing to standard post-surgical rehabilitation.	Both multimodal programs were home-based and consisted of moderate intensity aerobic and resistance exercise, nutrition counseling with daily whey protein supplementation and anxiety-reduction strategies.	Not explicitly described. Participants were given a pedometer to encourage daily walking. BCT: self-monitoring of behavior
McCourt et al. (2020) *Study Protocol* UK	60–75 patients with a diagnosis of myeloma	Intervention: exercise prehabilitation Control: usual care	To investigate the feasibility of a physiotherapist-led exercise intervention as an integral part of the myeloma autologous stem cell transplantation pathway at a UK tertiary center.	The exercise intervention comprises of partly supervised physiotherapist-led aerobic and resistance exercise including behavior change techniques to promote change in exercise behavior.	BCTs, to promote adherence to the intervention and behavior change were explicitly described as per the Taxonomy of BCTs and include: goal setting (behavior) problem solving, action planning, review behavior goal, discrepancy between current behavior and goal, feedback on behavior, self-monitoring of behavior, biofeedback, instruction on how to perform a behavior, information about health consequences, information about emotional consequences, demonstration of the behavior, behavioral practice/rehearsal, generalization of target behavior, graded tasks, credible source, pros and cons, adding objects to the environment, verbal persuasion about capability.
Carli et al. (2020) *Research Article* Canada	110 frail patients undergoing colorectal surgery	Prehabilitation group: *n* = 55 Rehabilitation group: *n* = 55	To assess the extent to which a prehabilitation program affects 30-day postoperative complications in frail patients undergoing colorectal cancer resection compared with postoperative rehabilitation.	Multimodal program involving exercise, nutritional, and psychological interventions initiated before (Prehab group) or after (Rehab group) surgery.	Not explicitly described Participants received counseling for smoking and alcohol cessation

a *Scientific publications from 2018 onwards only*.

b *Role of Behavioral science in terms of: use of theoretical frameworks in intervention development and evaluation; explicit mention of Behavior Change Techniques (BCTs) used in the intervention (where BCTs are not explicitly described as per the Taxonomy of BCTs they have been extracted); inclusion of Behavioral determinants as (outcome) variables, study aims directed at identifying Behavioral mechanisms or effective active ingredients; co-creation of the intervention*.

Importantly, some of the aforementioned studies also include qualitative process evaluations (Macleod et al., 2018; Brahmbhatt et al., 2020; McCourt et al., 2020). Brahmbhatt et al. (2020) present findings from interviews with participants who had participated in a home-based exercise prehabilitation program prior to breast cancer surgery. They appreciated the personalized exercise prescription which they could complete with ease, irrespective of pervious activity levels. In-person instruction on how to perform the exercise increased participant's confidence to exercise independently at home. Motivation, lack of time, and the weather were identified as barriers to participation. McCourt et al. (2020) plan to interview patients to explore experiences of involvement and patients who declined participation, to discuss experiences of being invited and their decision-making processes. This will afford important insights into the barriers and facilitators to involvement and enable refinement of future large-scale trials and/or services.

We encourage those designing new trials to include qualitative process evaluations by following published guidance (Moore et al., 2015). Not only does this allow exploration of patient experience, it can shed light on what worked for whom and in what context; vital data to support advancement and implementation of prehabilitation trials and services.

## Examples of Clinical Practice and Behavioral Science Input

Existing clinical prehabilitation services typically consist of advice on physical activity, diet, and anxiety or stress reduction techniques. Delivered either in a universal form for example videos or downloadable leaflets on the service provider's website, or personalized patient consultation with one or more members of the prehabilitation team. As seen in the research trial context, the place of program delivery varies. Table 2 summarizes key characteristics of some ongoing prehabilitation clinical programs. This list is not exhaustive, rather, a snapshot of international provision.

**Table 2:** Clinical prehabilitation services and behavioral science input.

**Location**	**Name**	**Brief description of service**	**Components**	**Evidence of behavioral science input**
Hospital Clinic Barcelona, Spain	PreHab	Supervised exercise, a mobile app-based personalized program to promote physical activity, dietary counseling and mindfulness sessions. In addition, psychological counseling is offered through specialist services as needed (Barberan-Garcia et al., 2020; Carli et al., 2020; PreHab, 2020)^a^	Physical, Nutritional, and Psychological (mindfulness)	Motivational interviewing is used as an underpinning delivery modality. The mobile app used to promote physical activity includes several behavior change strategies for example goal setting, self-monitoring, motivational tips, referral to smoking cessation services and alcohol services (Barberan-Garcia et al., 2020)
Manchester, UK	Prehab4Cancer	The service is based on ERAS+ (Prehab4Cancer, 2020) and includes Surgery School, and advice on how to increase daily exercise and perform breathing exercises, how to improve diet, advice on alcohol intake, and smoking reduction and management of psychological distress (Moore et al., 2017, 2020)	Physical, Nutritional, and Psychological	Not explicitly described The exercise component is delivered in community leisure centers aiming to induce long-term lifestyle behavioral change. Authors state inclusion of surgery school that uses “behavior change methodology” p3 (Moore et al., 2020), no details provided.
Montreal, Canada (Montreal General Hospital/McGill University)	Perioperative program (POP)	Home-based individualized exercise and diet prescription and teaching/practicing relaxation techniques. Supervised exercise is provided as needed. Smoking cessation support is included as needed (Barrett-Bernstein et al., 2019; Perioperative Program, 2020)^a^	Physical, Nutritional, and Psychological	Not explicitly described A primary goal of the psychological component was to “enhance and reinforce patients' motivation to comply with the exercise and nutrition aspects of the intervention” (Barrett-Bernstein et al., 2019)
Victoria, Australia (Peter MacCallum Cancer Centre)	Fit4Surgery	Personalized home-based and/or supervised gym exercise program, prescription of respiratory exercises, dietary advice, psychological support as needed and invitation to Surgery School (Peter MacCallum Cancer Centre, 2020; Tang et al., 2020)^a^	Physical, Nutritional, and Psychological	Not explicitly described The service development was informed by a patient experience-based co-design approach aiming to increase patient engagement (Tang et al., 2020). The psychological intervention includes discussions about ways to maintain motivation to carry out the activities throughout the prehabilitation period (Peter MacCallum Cancer Centre, 2020)
St Georges Hospital, London, UK	Get Set 4 Surgery	The service includes psychoeducation in the form of Surgery School (group psychoeducation) and access to the Macmillan Move More physical activity program (St George's University Hospital NHS Foundation Trust, 2020)	Physical, Psychoeducational	Not explicitly described
Imperial College Hospital, London, UK	PREPARE	A personalized exercise program and diet prescription, complemented by advice on respiratory exercises and psychological support as included in the service (Doganay and Moorthy, 2019; Imperial College Healthcare NHS Trust, 2020)	Physical, Nutritional, and Psychological	Not explicitly described The program includes use of exercise diaries for patients to monitor their progress and weekly review of progress by telephone contact from the exercise specialist. Progress with dietary changes is monitored and advice is adjusted as needed. The focus of psychological support is on improving self-efficacy. Behavior change support such as establishing short and long-term goals, providing feedback and connecting patients for peer support are employed (Doganay and Moorthy, 2019)
Maxima Medical Centre, Eindhoven, The Netherlands		Combination of fitness and strength training, nutritional support, psychological help and, if necessary, a smoking cessation process (van Rooijen et al., 2019b; Maxima Medical Centre, 2020)^b^	Physical, Nutritional, Psychological, and Smoking cessation	Not explicitly described The pilot RCT study for the multi-modal prehabilitation program included weekly phone calls with a specialist nurse to increase adherence to the program and included discussing coping mechanisms and encouraging “training perseverance” (p. 891). “Coaching” (p. 890) by a psychologist was also included although there are no details of what this entailed (van Rooijen et al., 2019b). It is unclear whether these elements were implemented in the service
Medway NHS Foundation Trust, UK	Kent and Medway Prehab	Personalized face-to-face or virtual (video/phone) exercise sessions, dietary and psychological support (Kent Medway, 2020; Wu et al., 2020)^a^	Physical, Nutritional, and Psychological	Not explicitly described Referral to smoking cessation and alcohol reduction services. The program is individualized taking into account people's preferences and values. Community setting makes service more accessible and increases the possibility of social support by family's and friends' (Wu et al., 2020)
South Tees, UK	PREP-WELL	Two supervised exercise sessions and home-based training. Respiratory exercise for those deemed high risk, dietary advice, nutrition support as appropriate and referral for mindfulness training or psychological counseling as needed (South Tees Hospital NHS Foundation Trust, 2020; Tew et al., 2020)^a^	Physical, Nutritional	Not explicitly described Referral to smoking cessation services, alcohol reduction services and psychological services

a *The referenced paper describes either a research study or a service improvement pilot that is a variant of the intervention in the service. The description of the intervention is based on the details available on public service websites. All efforts have been made to identify papers describing the closest variant of the published service*.

b *It is unclear from the available public information whether all elements of the pilot were implemented in the service*.

Mirroring the academic literature, most programs do not describe explicit consideration of behavioral science (though absence of evidence is not evidence of absence). However, there is emerging evidence of consideration of optimizing patient motivation and action. For example, the PreHab service in Barcelona, Spain, state patients receive a “motivational interview.” The POP program in Montreal, Canada recounts that “a primary goal of the psychological component was to enhance and reinforce patients' motivation to comply with the exercise and nutrition aspects of the intervention.” Others refer to ‘monitoring progress' and keeping “exercise diaries,” activities that support behavior change. However, few services explicitly describe involvement of team members with behavioral science expertise. Many prehabilitation services do however, signpost patients to specialist behavior change services such as alcohol reduction, smoking cessation, or weight management.

## Role of Surgery Schools

As well as clinical prehabilitation services as described above provision of preoperative education to groups of patients prior to major surgery (surgery school) has become increasingly common. In some clinical services this forms part of the prehabilitation program. A recent national survey undertaken by an author (IFJ) in collaboration with the Manchester prehabilitation leads, identified 32 active and planned surgery schools across the UK and Ireland. Historically these surgery schools focus on education, providing information on what to expect leading up to and following surgery and advisable lifestyle modifications. However, there is little evidence as to whether such schools catalyze behavior change. A recent publication reports 60% of patients attending surgery school in an hospital trust intended to change at least one lifestyle behaviors as a result of attending, and 46% reported doing so (Fecher-Jones et al., 2021). Although these results are encouraging, they highlight the long established “intention-behavior gap” with patients appreciating the potential benefit but without the skills or confidence to act (Rhodes and de Bruijn, 2013). These surgery schools present a unique opportunity to address this. Embedding BCTs that go beyond education and provide patients with skills and knowledge to enact new behaviors could have a powerful impact on many patients. Examples could be supporting realistic goal setting based on current activity levels and personal circumstances (e.g., caring responsibilities, physical environment, and access to facilities) and developing action plans that state specifically when, where and how a behavior will be performed. We therefore recommend professionals developing and delivering surgery schools work with behavioral science colleagues to embed these principles in their services.

## Discussion

Evidence of the benefits of cancer prehabilitation has burgeoned in the last few years and with it emerging clinical practices. There is increasing recognition of the importance of including strategies to enhance motivation and maximize compliance with programs. This is particularly important as programs move away from highly structured and supervised clinical environments to home and community-based settings. We encourage those developing new trials and services to collaborate with the behavioral science community to strengthen these efforts.

The key to successful behavior change interventions, like any other, is to understand the underlying determinants (in this case, that drive behavior) and what strategies are useful to influence them. Behavioral scientists can help address this; identifying underlying processes that impact on uptake and adherence and support the evaluation and refinement of these programs to maximize satisfaction and identify the most effective BCTs. Furthermore, working with end-users and iterative improvements based on their feedback is essential if programs are to be truly patient-centered and engagement maximized. Optimizing self-efficacy to engage in programs, as well as ensuring they are relevant to each patient are of notable importance.

It is also important to recognize credible concerns that complex interventions such as prehabilitation may inadvertently increase disparities between patients who do and do not engage. The demographic profile of cohorts involved in clinical trials in this area tend to over represent white, relatively young and well-educated populations. There is also a suggestion that clinicians can act as gatekeepers, choosing not to refer, for example, frail older adults to prehabilitation services or trials due to concerns that they may not be suitable or safe for these individuals. Arguably, these patients have the most to gain but may also need additional support to engage and adhere. It is therefore imperative that we strive collectively to increase inclusivity, working with all stakeholders and engaging with under-represented groups.

The prehabilitation community must focus on person-centered intervention development that enables patients to feel engaged and empowered. With more researchers and practitioners forging new collaborations with behavioral science colleagues to embed these principles in the development, optimization, and evaluation of new programs, we can deliver evidence and needs-based services that stand to provide enormous benefits to people with cancer.

## Data Availability Statement

The original contributions presented in the study are included in the article/supplementary material, further inquiries can be directed to the corresponding author/s.

## Author Contributions

CG drafted the manuscript. All authors (KB, SD, IF-J, MH, JV-S, CS, and CG) provided substantial intellectual contributions, reviewed, edited, and approved the final manuscripts.

## Conflict of Interest

The authors declare that the research was conducted in the absence of any commercial or financial relationships that could be construed as a potential conflict of interest.
